# Bile Acids Pneumonia: A Respiratory Distress Syndrome in Early-Term Neonates

**DOI:** 10.3390/jcm12206565

**Published:** 2023-10-17

**Authors:** Alessandro Perri, Maria Letizia Patti, Margherita Velardi, Annamaria Sbordone, Giorgia Prontera, Simona Fattore, Vito D’Andrea, Milena Tana, Giovanni Vento

**Affiliations:** 1 Department of Woman and Child Health and Public Health, Fondazione Policlinico Universitario Agostino Gemelli IRCCS, 00168 Rome, Italy; margherita.velardi@opbg.net (M.V.); annamaria.sbordone@guest.policlinicogemelli.it (A.S.); giorgia.prontera@guest.policlinicogemelli.it (G.P.); fattore.simona@guest.policlinicogemelli.it (S.F.); vito.dandrea@policlinicogemelli.it (V.D.); milena.tana@policlinicogemelli.it (M.T.); giovanni.vento@unicatt.it (G.V.); 2 Department of Woman and Child Health and Public Health, Child Health Area, Università Cattolica del Sacro Cuore, 00168 Rome, Italy

**Keywords:** neonatal distress syndrome, bile acids, lung ultrasound

## Abstract

Intrahepatic cholestasis of pregnancy (ICP) complicates among 0.2–2% of pregnancies and has been associated with adverse perinatal outcomes, including sudden stillbirth, meconium strained fluid, preterm birth, perinatal asphyxia, and transient tachypnea of the newborn. The diagnosis of “bile acids pneumonia” was previously proposed and a causative role of bile acids (BA) was supposed with a possible mechanism of action including surfactant dysfunction, inflammation, and chemical pneumonia. In the last few years, the role of lung ultrasound (LUS) in the diagnosis and management of neonatal respiratory distress syndrome has grown, and LUS scores have been introduced in the literature, as an effective predictor of the need for surfactant treatment among neonates with respiratory distress syndrome. We present four cases of infants born from pregnancies complicated by ICP, who developed respiratory distress syndrome early after birth. Lung ultrasound showed the same pattern for all infants, corresponding to a homogeneous alveolar–interstitial syndrome characterized by a diffuse coalescing B-line pattern (white lung). All infants evaluated require non-invasive respiratory support and in three cases surfactant administration, despite the near-term gestational age, with rapid improvement of respiratory disease and a good clinical outcome.

## 1. Introduction

Cholestasis of pregnancy complicates among 0.2–2% of pregnancies, with a higher incidence in Latina women in multiple gestations. The disease develops generally after 30 weeks of gestational age and is characterized by pruritus without rash, associated with elevated serum bile acids (BA) levels [1]. This condition resolves immediately after delivery. Although the disease is a benign medical condition for the mother, cholestasis of pregnancy has been associated with adverse perinatal outcomes, including sudden stillbirth (for placental vasospasm or cardiac arrhythmia), meconium strained fluid, preterm birth, perinatal asphyxia, and transient tachypnea of the newborn [2].

Bile acid levels correlate with the neonatal morbidity. Severe intrahepatic cholestasis (bile acids ≥ 100 micromole/L) is associated with worse perinatal outcomes [1]. Obstetrical management is usually based on intensive maternal–fetal surveillance and on a policy of near-term birth induction [3]. The frequency of obstetrical checks is not associated with a lower risk of adverse outcomes [1]. Treatment with ursodeoxycholic acid (UDCA) has been effective in reducing pruritus and improving liver function [4] but a recent placebo-controlled trial showed that UDCA did not improve the analyzed perinatal outcomes [5].

In 2004 Zecca et al. [6] described a severe respiratory distress syndrome (RDS) in term or near-term neonates from mothers with intrahepatic cholestasis of pregnancy (ICP). The diagnosis of “bile acids pneumonia” was proposed and a causative role of BAs was supposed with a possible mechanism of action including surfactant dysfunction, inflammation, and chemical pneumonia. A successive study of the same group [7] showed that BAs were present in the bronchoalveolar lavage (BAL) of ICP neonates with RDS and were absent in the control group. BA in the lung could enhance secretory phospholipase A2 in the alveoli, which diminishes surfactant levels. An alternative supposed pathway was a direct “chemical” damage produced by BA on type II pneumocytes.

The application of lung ultrasound (LUS) in neonatology has increased during the last decade, as an accurate, reliable, quick, easy-to-use, real-time, low-cost, and radiation-free imaging modality [8,9,10]. Several related studies have demonstrated that LUS produces high sensitivity, specificity, and positive and negative predictive values in neonatal RDS diagnosis, and to predict NICU admission and surfactant administration [10,11,12,13]. In 2015, Brat et al. validated in neonatal medicine a classification system used in adults. The neonatal-adapted LUS score is a progressive numerical score assigned to a lung image series that reflects lung aeration, correlates with oxygenation and may predict surfactant administration [14].

In the last years, LUS scores have been introduced in the literature, as an effective predictor of the need for surfactant treatment among neonates with RDS [15,16,17,18,19]. Moreover, subpleural consolidations, consolidations > 1 cm, dynamic bronchogram, focal-B lines, pleural line abnormalities and pleural effusion have been shown to be characteristics of pneumonia in infants and children [20]. However, bile acid pneumonia has never been clearly characterized using lung ultrasound, as the findings can vary from the presence of consolidations to the more generic pattern of white lung, which is characteristic of surfactant deficiency disease. Furthermore, as described in the previously mentioned studies, surfactant deficiency could play a causative role in bile acid pneumonia.

We describe four cases of respiratory distress syndrome in near-term newborns whose mothers suffered from intrahepatic cholestasis of pregnancy (ICP). We present the findings in lung ultrasound. Our hypothesis is that this condition could be better and more easily characterized by this technique compared to classical radiography. Moreover, with a timely diagnosis, the management could be addressed accordingly.

## 2. Materials and Methods

This study was conducted between September 2021 and July 2022, at the Neonatal Intensive Care Unit (NICU) of Fondazione Policlinico Universitario “A. Gemelli” IRCCS (Rome, Italy). We evaluated four term/late-preterm infants born from a pregnancy complicated by ICP, and who developed respiratory distress after birth that required non-invasive ventilation.

A lung ultrasound was obtained at the admission (between 2 and 6 h after birth), by a trained neonatologist. The probe was a 12 MHz linear one, and the sonograms were taken with a General Electric LOGIQ E sonographer. The whole chest wall was scanned in six regions: the anterior, lateral and posterior regions for each side. Boundaries were the anterior and posterior axillary lines. Oral 33% glucose was used as a measure to prevent patient discomfort. When required, endotracheal surfactant was administered according to current European Consensus Guidelines on the management of respiratory distress syndrome [21], using INSURE technique, intravenous premedication with atropine and fentanest was administered when intubation was needed.

The pregnancy data and demographic, clinical and LUS information were extracted from patient records (Digistat^®^) and included: gestational age, birth weight, mode of delivery, APGAR score, BA level in pregnancy, age of surfactant administration, LUS score, duration of CPAP after surfactant administration and duration of hospitalization.

Written informed consent was obtained from parents for all infants.

## 3. Cases in Study

The first examined case was an early-term male neonate, (gestational age of 37 + 2 weeks, weighing 3665 g), born by an induced vaginal delivery. The mother had a diagnosis of intrahepatic cholestasis of pregnancy (ICP) at 21 weeks of gestation and reported a maximum level of bile acids of 20.5 µmol/L at a gestational age of 36 + 2 weeks. She was in therapy with ursodeoxycholic acid and had no symptoms associated with the condition. Fetal ultrasound scans performed during pregnancy were reported as normal except for polyamnios. The maternal infectious serologies for TORCH indicated no active infection and vaginal and rectal swabs for Streptococcus Agalactiae were negative, no signs of chorioamnionitis were reported. At birth, the newborn had a good cardiorespiratory adaptation with an Apgar score of 9 in the first minute and 10 in the fifth minute. At 3 h and 10 min of life, the baby showed respiratory distress (Silverman score 3-4) and was admitted to the Neonatal Intensive Care Unit. Respiratory assistance with Continuous Positive Airway Pressure (CPAP) with oxygen supplementation was started to maintain good vital parameters. A lung ultrasound performed approximately at 5 h and 30 min of life demonstrated findings of bilateral white lung and pleural thickening with interruption spots, especially at the basal level. A diagnosis of respiratory distress syndrome (RDS) with a LUS of 11 was made. At 31 h of life, a second lung ultrasound was performed, reporting a bilateral thickening of the pleural line and a white lung pattern in all regions, associated with small hypoechoic lesions in the apical and basal areas on the mid-clavicular line, suggestive of unventilated areas. The LUS score was stable at 11, with an oxygen supplementation >30%. At 34 h of life, a dose of endotracheal surfactant at 200 mg/kg was administered using the INSURE technique. The BAL collected during the intubation procedure, as per internal protocol for each intubation, was negative. No other infective cultures were collected and no antibiotic therapy was started, considering the absence of prenatal infectious risk factors and the clinical improvement after surfactant administration. The baby needed oxygen supplementation for 39 h, while the non-invasive respiratory assistance was gradually weaned and stopped after 96 h. The neonate was discharged at home in good general condition on DOL (day of life) 8.

The second case is about an early-term male neonate (gestational age 37 + 5 weeks), born by an elective cesarean section with a neonatal weight adequate for gestational age (3500 g). The mother had a diagnosis of ICP and reported a maximum level of bile acids of 18 µmol/L. She was in therapy with ursodesossicolic acid and ademetionine with no associated symptoms. Fetal ultrasound scans performed during pregnancy were reported as normal. The maternal infectious serologies for the TORCH complex indicated no active infection. Due to the positivity of the vaginal swab for Streptococcus Agalactiae performed six days before delivery, adequate intrapartum antibiotic therapy was administrated to the woman. No signs of chorioamnionitis were reported. At birth, the newborn had a good cardiorespiratory adaptation with an Apgar score of 9 at both the first and fifth minute. At around 1 h and 30 min of life, the baby showed respiratory distress with oxygen requirements up to 30%, requiring admission to the Neonatal Intensive Care Unit. Respiratory assistance with CPAP and oxygen supplementation was started to maintain good cardiorespiratory parameters. At approximately 2 h and 30 min of life, a lung ultrasound was performed with findings of bilateral white lung and pleural thickening, LUS score of 12. At 7 h and 30 min of life, a dose of endotracheal surfactant at 200 mg/kg was administered using the INSURE technique. The infectious cultures were performed in consideration of prenatal risk factors: bronchoalveolar lavage (BAL) collected during the intubation procedure resulted positive for *Pseudomonas* spp., while blood and urine cultures resulted negative. Antibiotic treatment with teicoplanine and amikacine was administered for 6 days. A lung ultrasound at 27 h of life reported an improvement of the pulmonary pattern with the persistence of the pleural line thickening and the presence of coalescent B lines only on the basal regions. During the hospitalization, the respiratory distress and general conditions progressively improved. The supplementation of oxygen was stopped after 14 h and respiratory assistance with CPAP was progressively reduced and stopped after approximately 103 h. The neonate was discharged home in good general condition on DOL 9.

The third case refers to a male late preterm (gestational age 35 + 1 weeks) weighing 2910 g at birth, adequate for gestational age. The baby was born from an elective cesarean section performed for previous cesarean section, contractile activity and symptomatic ICP (asthenia, nausea and vomiting, treated with esomeprazole and severe itch treated with chlorfenamine). The mother was diagnosed with ICP at around 23 weeks of gestation and reported a maximum level of bile acids of 112 µmol/L at a gestational age of 33 + 0, in therapy with ursodesossicolic acid and ademetionine. A prophylaxis for fetal pulmonary maturation with betamethasone was administered to the woman at the correct time. Fetal ultrasound scans performed during pregnancy were reported as normal. The maternal infectious serologies for the TORCH complex indicated no active infection. Vaginal and rectal swabs were negative for Streptococcus Agalactiae. No signs of chorioamnionitis were reported. At birth, the newborn had a good cardiorespiratory adaptation with an Apgar score of 8 in the first minute and 9 in the fifth minute. At around 1 h and 20 min of life, the baby appeared to be in respiratory distress and requested a supplementation of oxygen up to 28%. The baby was then admitted to the Neonatal Intensive Care Unit and non-invasive respiratory assistance was started with CPAP, to maintain good cardiorespiratory parameters. At approximately 3 h and 30 min of life, a lung ultrasound was performed with findings of bilateral white lung and pleural thickening, LUS score of 12. At 25 h of life, the newborn received a dose of endotracheal surfactant at 200 mg/kg using the INSURE technique. Bronchoalveolar lavage (BAL) collected during the intubation procedure was negative. No antibiotic therapy was administrated. A lung ultrasound was repeated on DOL 6, showing a normal pulmonary pattern, LUS 0. During the hospitalization, the respiratory distress and general conditions progressively improved. The baby needed oxygen supplementation for 32 h. Respiratory assistance with CPAP was progressively reduced and stopped after approximately 62 h. The neonate was discharged home in good general condition on DOL 7.

The last case is about a male neonate born at 38 + 3 weeks of gestational age from an elective cesarean section. The neonatal weight was 3.420 g, adequate for gestational age. The mother showed an increased level of bile acids (10.2 µmol/L) and transaminases (GPT 246 UI/L) in the last week of gestation. Maternal serologies for the TORCH complex indicated no active infection. Vaginal and rectal swabs were negative for Streptococcus Agalactiae, but the vaginal swab resulted in positive for Candida Albicans. Neither maternal fever nor other signs of chorioamniotis were reported. Fetal ultrasound scans performed during pregnancy were reported as normal. At birth, the newborn had a good cardiorespiratory adaptation and an Apgar score of 9 in the first minute and 10 in the fifth minute. At 20 min of life, the baby appeared to be in respiratory distress with oxygen requirements increased to 40%, requiring respiratory assistance with CPAP to maintain adequate cardio-respiratory parameters. The baby was admitted to the Neonatal Intensive Care Unit. At approximately 2 h and 15 min of life, a lung ultrasound was performed, with findings of pleural thickening bilaterally and a sign of pleural interruption associated with an ipoechoic area in the basal right lung, B lines were in the number of three or more, LUS was 6. No cultures were performed and neither antibiotic therapy nor surfactant was administrated, considering the respiratory improvement after CPAP was started with no oxygen supplementation needed. A lung ultrasound scan was repeated at 75 h of life and it resulted to be normal with a resolution of the ipoechoic lesion previously described at the base of the right lung. During the hospitalization, the respiratory distress progressively improved and respiratory assistance with CPAP was progressively reduced and stopped after approximately 21 h. The neonate was discharged home in good general condition on DOL 11.

Table 1 summarizes the characteristics of the four patients.

Of the four infants examined, three were born early term (37–38 weeks of gestational age -GA-), and one of them was born late preterm (GA 35 weeks); means of birth weight were 3373 ± 231 g.

LUS score was suggested for surfactant administration in three of the four cases and the mean age at surfactant administration was 29.5 ± 4.5 h.

After surfactant therapy, the mean duration of nCPAP was 68 ± 23.3 h, and the neonates were discharged at an average of 8.7 ± 1.2 days of life.

## 4. Discussion

**Figure 1:**
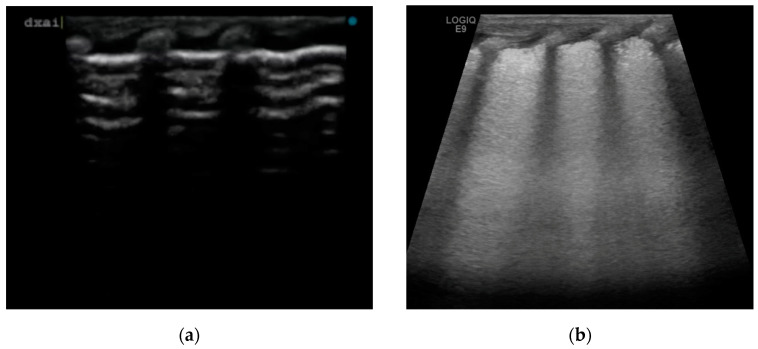
Lung ultrasound images: (**a**) normal lung, with A-line pattern; (**b**) diffuse coalescing B-line pattern (white lung), with pleural thickening with some interruption spots and the disappearance of A-line.

We have described four cases of respiratory distress syndrome in babies born at term or near term from mothers with a diagnosis of ICP during pregnancy, treated with surfactant administration and with a rapid resolution of the respiratory distress symptoms, based on a supposed causative role of BAs in surfactant dysfunction. The diagnosis of ICP was made in the second trimester of pregnancy for two of the four cases, and at 37 weeks of GA in patient 4. All the women were Caucasian. In all patients, we observed early onset of signs and symptoms of respiratory distress after birth, with variable severity. The neonates required non-invasive support with nasal cannula continuous positive airway pressure (CPAP) at the admission in NICU. The lung ultrasounds performed in the first six hours of life, showed the same features for all patients, with a homogeneous alveolar–interstitial syndrome characterized by a diffuse coalescing B-line pattern (white lung), with pleural thickening with some interruption spots and the disappearance of A-line (Figure 1b). None of them had subpleural consolidation. LU scores were predictive of surfactant administration in three of the four cases.

ICP is the most common liver disorder in pregnancy, which can begin typically in the second trimester and resolves after delivery in the puerperium. The serum concentration of BA in the fetus, which synthesizes a large amount of BA, is controlled by a placental transfer system in the fetus-to-mother direction [22]. BA can be injurious to the lungs. Animal models and studies on human adults allowed us to show surfactant dysfunction, inflammation and chemical pneumonia as possible mechanisms [7]. In 2007, Zecca et al. [23] demonstrated, in a cohort of infants delivered from ICP pregnancies, that BA was present in the BAL of ventilated neonates with RDS but was absent in neonates ventilated for other reasons and in infants with no lung disease. BA in the lung could enhance secretory phospholipase A2, which diminishes surfactant levels. An alternative mechanism may be a direct “chemical” damage produced by BA on type II pneumocytes, throughout the intracellular Ca++ increment.

Although BA levels during pregnancy may influence neonatal outcomes [1], a cut-off level to predict neonatal RDS has a limited clinical value before delivery.

In our cases, we made the clinical diagnosis of BA pneumonia because a causative role of BA was likely to be the main one. In fact, the classic hyaline membrane disease was difficult to diagnose in near-term infants. All the infants had a normal Apgar score, and meconium aspiration syndrome was excluded for the absence of meconium staining of the amniotic fluid. Infective pneumonia was excluded by negative cultures when performed, or the rapid resolution of symptoms after surfactant administration and brief need for ventilatory assistance. In case 3, *Pseudomonas* spp. was isolated also in the superficial surveillance swab, thus assuming a colonization, also for the rapid resolution of the symptoms. Neonatal transient tachypnea was excluded by the absence of a typical pattern in the lung ultrasound (double lung point) and by the progressive worsening of respiratory symptoms over time, prior to surfactant administration.

In our center, we perform a lung ultrasound on all infants with respiratory distress syndrome by internal protocol. In these cases, we found a lung ultrasound pattern characterized by a “white lung” and thickening or interruptions of pleural line, that was similar to the pattern usually described for the RDS [10,24].

In three of the four cases, the infants met the clinical criteria for surfactant administration, according to European Consensus Guidelines on the management of respiratory distress syndrome [21]. Moreover, as previously mentioned, the LUS score is well correlated with oxygenation status in both term and preterm neonates, regardless of the underlying respiratory condition, and may help to correctly identify the babies that need surfactant administration [14]. In all the cases treated with surfactant, the LUS score was high (case 1 LUS score 11, case 2 LUS score 12, case 3 LUS score 12).

The response to endotracheal surfactant therapy was optimal in all three infants, with a rapid clinical improvement (early suspension of the CPAP) and a progressive normalization of the lung ultrasound.

For case 4, we assumed that the late onset of ICP in the pregnancy had a minor impact on the development of lung disease.

Our clinical approach in all cases was targeted to early surfactant replacement therapy, despite the onset of respiratory distress in a population of near-term infants. The clinical decision was supported by the lung ultrasound, which showed, especially in the three most severe cases, a white lung pattern and higher LUS scores. The optimal response to surfactant therapy is in line with the pathophysiological hypothesis of diminished surfactant levels in infants born to mothers affected by ICP. Moreover, the early administration of surfactant allowed the rapid resolution of respiratory symptoms, avoiding unnecessary antibiotic therapy, and therefore a shorter duration of hospitalization.

This study, however, has several limitations including the small number of patients and the retrospective design, associated with the rarity of the disease. The small number of patients depends on our intention to select only those who had no additional risk factors for respiratory disease. Moreover, according to our clinical records (about 4000 births/year), not so many late preterm or term babies whose mothers were diagnosed with ICP showed respiratory distress symptoms. A prospective study would be necessary in the future, with the aim to analyze the levels of BAs in the BAL of symptomatic infants born from mothers with ICP, and the possible correlation with the response to surfactant administration.

## 5. Conclusions

In our small case series, in the babies we studied, we found an ultrasound picture comparable to hyaline membrane disease. Moreover, despite the gestational age of the patients, we treated the pathology with surfactant administration, obtaining an excellent clinical response and short resolution times of the respiratory symptoms. Based on our experience, we suggest a high level of attention during the first hours of life in all newborn infants born from pregnancies complicated by ICP. We suggest focusing on the onset of any sign and symptom of RDS and, despite the term GA, we suggest considering an early treatment with surfactant therapy, based on the possible mechanism of action of BAs, including surfactant dysfunction, in order to improve the respiratory outcome and reduce the hospitalization.

## Figures and Tables

**Table 1:** Summary of patient characteristics. (CS = cesarean section; GA = gestational age; ICP = intrahepatic cholestasis of pregnancy; BA = bile acids; NICU = neonatal intensive care unit; LUS = lung ultrasound; CPAP = Continuous Positive Airway Pressure; BAL = bronchoalveolar lavage).

	Patient 1	Patient 2	Patient 3	Patient 4
GA, weeks/days	37.2	37.5	35.1	38.3
Mode of delivery	Vaginal	CS	CS	CS
Birth weight, g	3665	3500	2910	3420
Apgar score 1’-5’	9-10	9-9	8-9	9-10
GA at ICP diagnosis	21	-	23	37
BA level, µmol/L	20.5	18	112	10.2
Ursodexoxycholic therapy	Yes	Yes	Yes	no
Vaginal cultures during pregnancy	negative	Streptococcus Agalactiae	negative	negative
Intrapartum antibiotic profilaxis	-	adequate	-	-
Time of onset of respiratory distress	3 h 10 min	1 h 30 min	1 h 20 min	20 min
Respiratory support in NICU	CPAP	CPAP	CPAP	CPAP
FiO_2_ prior to surfactant administration	>0.30	>0.30	0.30	0.21
Age of surfactant administration, hours	34	7.30	25	/
LUS score	11	12	12	6
Duration of CPAP after surfactant administration, hours	39	103	62	/
Infectious cultures performed	none	BAL (*Pseudomonas* spp.), blood (negative), urine (negative)	BAL (negative)	none
Antibiotics administration	-	yes (7 days)	-	-
Duration of hospitalization, days	8	9	7	11

## Data Availability

The data presented in this study are available on request from the corresponding author.

## References

[1] Herrera C.A., Manuck T.A., Stoddard G.J., Varner M.W., Esplin S., Clark E.A.S., Silver R.M., Eller A.G. (2018). Perinatal outcomes associated with intrahepatic cholestasis of pregnancy. J. Matern. Fetal Neonatal Med..

[2] Williamson C., Hems L.M., Goulis D.G., Walker I., Chambers J., Donaldson O., Swiet M., Johnston D.G. (2004). Clinical outcome in a series of cases of obstetric cholestasis identified via a patient support group. BJOG.

[3] Zecca E., De Luca D., Barbato G., Marras M., Tiberi E., Romagnoli C. (2008). Predicting respiratory distress syndrome in neonates from mothers with intrahepatic cholestasis of pregnancy. Early Hum. Dev..

[4] Luo M., Tang M., Jiang F., Jia Y., Chin R.K.H., Liang W., Cheng H. (2021). Intrahepatic Cholestasis of Pregnancy and Associated Adverse Maternal and Fetal Outcomes: A Retrospective Case-Control Study. Gastroenterol. Res. Pract..

[5] Chappell L.C., Bell J.L., Smith A., Linsell L., Juszczak E., Dixon P.H., Chambers J., Hunter R., Dorling J., Williamson C. (2019). Ursodeoxycholic acid versus placebo in women with intrahepatic cholestasis of pregnancy (PITCHES): A randomised controlled trial. Lancet.

[6] Zecca E., Costa S., Lauriola V., Vento G., Papacci P., Romagnoli C. (2004). Bile acid pneumonia: A “new” form of neonatal respiratory distress syndrome?. Pediatrics.

[7] Zecca E., De Luca D., Baroni S., Vento G., Tiberi E., Romagnoli C. (2008). Bile acid-induced lung injury in newborn infants: A bronchoalveolar lavage fluid study. Pediatrics.

[8] Raimondi F., Yousef N., Migliaro F., Capasso L., De Luca D. (2021). Point-of-care lung ultrasound in neonatology: Classification into descriptive and functional applications. Pediatr. Res..

[9] Escourrou G., De Luca D. (2016). Lung ultrasound decreased radiation exposure in preterm infants in a neonatal intensive care unit. Acta Paediatr..

[10] Wang J., Wei H., Chen H., Wan K., Mao R., Xiao P., Chang X. (2022). Application of ultrasonography in neonatal lung disease: An updated review. Front. Pediatr..

[11] Kurepa D., Zaghloul N., Watkins L., Liu J. (2018). Neonatal lung ultrasound exam guidelines. J. Perinatol..

[12] Jagła M., Grudzień A., Starzec K., Tomasik T., Zasada M., Kwinta P. (2019). Lung ultrasound in the diagnosis of neonatal respiratory failure prior to patient transport. J. Clin. Ultrasound.

[13] Srinivasan S., Aggarwal N., Makhaik S., Jhobta A., Kapila S., Bhoil R. (2022). Role of lung ultrasound in diagnosing and differentiating transient tachypnea of the newborn and respiratory distress syndrome in preterm neonates. J. Ultrason..

[14] Brat R., Yousef N., Klifa R., Reynaud S., Shankar Aguilera S., De Luca D. (2015). Lung Ultrasonography Score to Evaluate Oxygenation and Surfactant Need in Neonates Treated With Continuous Positive Airway Pressure. JAMA Pediatr..

[15] Raschetti R., Yousef N., Vigo G., Marseglia G., Centorrino R., Ben-Ammar R., Shankar-Aguilera S., De Luca D. (2019). Echography-Guided Surfactant Therapy to Improve Timeliness of Surfactant Replacement: A Quality Improvement Project. J. Pediatr..

[16] Pillers D.A. (2019). Lung ultrasound may allow for timelier surfactant administration and reduce radiograph use. J. Pediatr..

[17] De Martino L., Yousef N., Ben-Ammar R., Raimondi F., Shankar-Aguilera S., De Luca D. (2018). Lung Ultrasound Score Predicts Surfactant Need in Extremely Preterm Neonates. Pediatrics.

[18] Gregorio-Hernández R., Arriaga-Redondo M., Pérez-Pérez A., Ramos-Navarro C., Sánchez-Luna M. (2020). Lung ultrasound in preterm infants with respiratory distress: Experience in a neonatal intensive care unit. Eur. J. Pediatr..

[19] Vardar G., Karadag N., Karatekin G. (2021). The Role of Lung Ultrasound as an Early Diagnostic Tool for Need of Surfactant Therapy in Preterm Infants with Respiratory Distress Syndrome. Am. J. Perinatol..

[20] Tusor N., De Cunto A., Basma Y., Klein J.L., Meau-Petit V. (2021). Ventilator-associated pneumonia in neonates: The role of point of care lung ultrasound. Eur. J. Pediatr..

[21] Sweet D.G., Carnielli V., Greisen G., Hallman M., Ozek E., Te Pas A., Plavka R., Roehr C.C., Saugstad O.D., Simeoni U. (2019). European Consensus Guidelines on the Management of Respiratory Distress Syndrome-2019 Update. Neonatology.

[22] Ushijima K., Kimura A., Inokuchi T., Yamato Y., Maeda K., Yamashita Y., Nakashima E., Kato H. (2001). Placental transport of bile acids: Analysis of bile acids in maternal serum and urine, umbilical cord blood, and amniotic fluid. Kurume Med. J..

[23] Oelberg D.G., Downey S.A., Flynn M.M. (1990). Bile salt-induced intracellular Ca++ accumulation in type II pneumocytes. Lung.

[24] Lichtenstein D.A., Mauriat P. (2012). Lung Ultrasound in the Critically Ill Neonate. Curr. Pediatr. Rev..

